# Psychosomatic Profiles and Their Association with Health Behaviors in Patients with Inflammatory Bowel Disease (IBD) and Low Disease Activity

**DOI:** 10.3390/jcm14227944

**Published:** 2025-11-09

**Authors:** Graziano Gigante, Sara Gostoli, Chiara Dettori, Maria Montecchiarini, Alessia Urgese, Anna M. Polifemo, Francesco Guolo, Francesco Ferrara, Vincenzo Cennamo, Chiara Rafanelli

**Affiliations:** 1 Department of Psychology “Renzo Canestrari”, University of Bologna, Viale Berti Pichat 5, 40127 Bologna, BO, Italy; graziano.gigante2@unibo.it (G.G.); sara.gostoli2@unibo.it (S.G.); chiara.dettori@studio.unibo.it (C.D.); montecchiarinimaria@gmail.com (M.M.); alessia.urgese@gmail.com (A.U.); 2 Gastroenterology and Interventional Endoscopy Unit, Bellaria Hospital, AUSL Bologna, Via Altura 3, 40139 Bologna, BO, Italy; a.polifemo@ausl.bologna.it (A.M.P.); f.ferrara@ausl.bologna.it (F.F.); vincenzo.cennamo@ausl.bologna.it (V.C.); 3 Division of Cardiology, Bellaria Hospital, AUSL Bologna, Via Altura 3, 40139 Bologna, BO, Italy; francesco.guolo@studio.unibo.it

**Keywords:** Diagnostic Criteria for Psychosomatic Research (DCPR), health behaviors, Inflammatory bowel disease (IBD), psychosomatic syndrome

## Abstract

**Background/Objectives**: Inflammatory bowel diseases (IBDs) are chronic conditions with significant psychological comorbidities. Research on subclinical psychosomatic syndromes in this category of patients remains limited. This study aimed to identify distinct psychosomatic profiles using the Diagnostic Criteria for Psychosomatic Research—Revised (DCPR-R) framework and to examine their associations with health behaviors. **Methods**: A total of 111 adult IBD patients in clinical remission or with low disease activity were enrolled and underwent a comprehensive assessment of sociodemographic and clinical variables, psychosomatic syndromes and health behaviors. Hierarchical clustering using complete linkage and Jaccard distance was performed on two subsets of common psychosomatic syndromes in this sample. Associations between psychosomatic syndromes and profiles and health behaviors were examined using multiple regression models, controlling for IBD severity and applying Benjamini–Hochberg correction. Analyses were conducted according to a pre-registered protocol. **Results**: Six distinct clusters were identified: irritability (9.3%), pure allostatic overload (18.7%), pure alexithymia (26.7%), overwhelmed type A behavior (12%), subclinical depressive mood (13.3%), and alexithymic type A behavior (26.7%). Pure alexithymia patients showed higher smoking odds (*OR* = 3.85, *p* = 0.033) and overwhelmed type A behavior patients showed less frequent physical activity (*OR* = 0.27, *p* = 0.018). For individual syndromes, irritable mood was associated with lower alcohol consumption (*OR* = 0.29, *p* = 0.035) while type A behavior was associated with higher consumption (*OR* = 1.87, *p* = 0.030). However, all associations became non-significant after false discovery rate correction. **Conclusions**: IBD patients with low disease activity exhibit distinct psychosomatic profiles. While specific syndromes and profiles showed trends toward certain health behaviors, associations were not robust to multiple comparison correction. Psychosomatic profiling may inform personalized clinical approaches, though larger studies are needed to establish definitive associations with health behaviors.

## 1. Introduction

Inflammatory bowel diseases (IBDs), which include Crohn’s disease (CD) and ulcerative colitis (UC), are chronic conditions characterized by an alternating course of active inflammation and remission. These disorders represent a significant burden for patients’ physical health and overall quality of life [1]. The clinical picture is often complicated by extraintestinal manifestations and a high prevalence of psychological distress [2]. In particular, psychiatric comorbidities such as anxiety and depression are well documented in IBD populations and are known to influence disease progression and treatment adherence [3,4].

In addition to diagnosable psychiatric disorders, recent advances in psychosomatic medicine have emphasized the role of subclinical syndromes: clusters of psychological symptoms that, while not meeting DSM criteria for a diagnosis, still exert a significant influence on clinical outcomes and patient functioning [5]. The Diagnostic Criteria for Psychosomatic Research—Revised (DCPR-R) provide a validated framework which allows to identify these syndromes, including allostatic overload, demoralization, alexithymia, health anxiety, and various maladaptive illness behaviors [6]. These constructs have shown substantial clinical relevance across a range of medical populations, including patients with cardiovascular disease [7,8], cancer [9,10], and chronic pain [11].

Despite this growing body of evidence, research on DCPR-R syndromes in IBD remains scarce. A recent longitudinal study by Gostoli et al. [12] found that approximately 71% of IBD outpatients met criteria for at least one psychosomatic syndrome at baseline, and 76% at follow-up. The most persistent syndromes over time were allostatic overload, type A behavior, and demoralization. However, no studies to date have examined the prevalence and distribution of DCPR-R syndromes specifically in patients with low disease activity, nor has their potential role in shaping lifestyle-related behaviors been explored.

This represents a critical gap in the literature, as maintaining remission in IBD relies heavily on lifestyle factors such as regular physical activity, balanced nutrition, limited alcohol consumption, and smoking cessation [13,14]. Nevertheless, adherence to these recommendations remains inconsistent among patients—as evidenced, for instance, by Çelik et al. [15], who found that only around 22% of IBD patients showed strong adherence to a Mediterranean-style diet. In other chronic illness populations, psychosomatic syndromes have been linked to diminished motivation for self-care and unhealthy behaviors. For instance, demoralization—a state of helplessness and loss of purpose—has been associated with physical inactivity, avoidance, and poorer psychosocial adjustment [16,17]. Similarly, alexithymia has been implicated in maladaptive coping strategies such as alcohol use and smoking, possibly as a way to regulate unprocessed emotions [18,19]. It is plausible that similar mechanisms operate in IBD, yet no studies have directly investigated these associations within a DCPR framework.

The present study aims to address this gap by conducting a psychosomatic profiling of patients with IBD in clinical remission or low disease activity, based on the DCPR-R framework. The first aim is to describe the prevalence and clustering of DCPR-R syndromes in this population. The second aim is to examine whether the presence of one or more psychosomatic syndromes is associated with lifestyle-related behaviors (i.e., physical activity, diet, alcohol consumption, smoking), controlling for disease-specific variables. By integrating a psychosomatic assessment into the clinical evaluation of IBD patients, the study seeks to contribute to a more comprehensive understanding of behavioral determinants in IBD management, in order to shape future targeted interventions.

The study is guided by the following hypotheses:

1. IBD patients with low disease activity will show distinct psychosomatic syndrome profiles that can be grouped into meaningful clusters.
2. After controlling for disease-related variables, the presence of one or more psychosomatic syndromes is associated with:
  - a reduction in the frequency and intensity of physical activity;
  - poorer dietary habits;
  - higher alcohol consumption;
  - more detrimental smoking behaviors.

## 2. Materials and Methods

### 2.1. Participants and Procedure

Patients were consecutively recruited within the Gastroenterology Unit of the Local Unit Health (AUSL) in Bologna, Italy, during routine follow-up consultations conducted between December 2018 and January 2020. Inclusion criteria were: (a) age between 18 and 75 years, (b) confirmed diagnosis of Crohn’s disease or ulcerative colitis according to ECCO-ESGAR guidelines [20], and (c) disease in remission or low activity. Disease activity was determined by a gastroenterologist during an outpatient visit, through the assessment of the most recent endoscopic exam, fecal examination, blood tests with specific reference to C-reactive protein and complete blood count, and assessment of patient-reported symptomatology (e.g., gastrointestinal symptoms, number of daily bowel movements). For Crohn’s disease, evaluation of intestinal ultrasound and/or MR enterography (when available) was conducted as well. Exclusion criteria were: (a) current psychotic or cognitive disorders preventing reliable interview participation, (b) lack of fluency in Italian, or (c) refusal to provide informed consent.

After signing the informed consent form, patients underwent a structured clinical interview conducted by two trained psychologists with prior experience in the application of the study instruments and adherence to standardized administration protocols was ensured throughout. The average interview duration was approximately 45 min. All procedures conformed to the ethical standards of the institutional and national research committee and the 1975 Helsinki Declaration, as revised in 2008.

### 2.2. Assessment

All assessments were conducted through structured face-to-face interviews during scheduled outpatient visits. The interviews were carried out in a quiet and private room within the Gastroenterology Unit.

The assessment protocol included the following components: (1) the structured interview for the Diagnostic Criteria for Psychosomatic Research—Revised [6] to evaluate the presence of psychosomatic syndromes; (2) a selection of items from the GOSPEL Questionnaire [21] focused on lifestyle-related behaviors, including physical activity, dietary patterns, alcohol consumption, and smoking; and (3) a brief data sheet developed ad hoc for the collection of sociodemographic and clinical information.

All information collected during the interviews was entered into a secure, anonymized database immediately after each session. Data collection procedures complied with ethical standards and were approved by the local institutional review board.

### 2.3. Materials

#### 2.3.1. Sociodemographic and Clinical Variables

Sociodemographic information was collected during the clinical interview and included patient age, gender, education level, employment status, and marital status.

Clinical variables were obtained through the integration of medical records and patient self-reports. These included the type of diagnosis (i.e., Crohn’s disease and ulcerative colitis), the total duration of illness in years, and current pharmacological treatments. In order to account for disease-related variability in analyses, a composite covariate termed IBD Severity was constructed. Previous literature has presented different methods for evaluating IBD severity, based on the assessment of multiple clinical, pharmacological, and subjective factors [22,23]. Given the differences in the concept of disease severity in patients with UC and CD [22], and given the limited availability of the measurements required for validated assessment of disease severity in the dataset used for this secondary analysis, a simplified clinical marker was constructed using available variables that are components of validated severity indices and are associated with disease severity in the literature [22,23]. This variable was coded as “severe” when patients were either undergoing immunosuppressive therapy—such as corticosteroids or azathioprine—or had a documented history of intestinal surgery due to IBD. If neither of these conditions were present, the patient was classified as “not severe”.

#### 2.3.2. Psychosomatic Syndromes

Psychosomatic syndromes were assessed using the Italian version of the semi-structured interview based on the Diagnostic Criteria for Psychosomatic Research—Revised (DCPR-R-SSI) [6]. This interview evaluates the presence of 14 psychosomatic syndromes grouped across four clinical domains: stress (i.e., allostatic overload), illness behaviors (i.e., health anxiety, disease phobia, hypochondriasis, thanatophobia, illness denial, persistent somatization, conversion symptoms, anniversary reaction), psychological manifestations (i.e., somatic symptoms secondary to a psychiatric disorder, demoralization, irritable mood), and personality attributes (i.e., type A behavior, alexithymia). Each syndrome is identified through a set of dichotomous items (yes/no) scored according to specific diagnostic rules.

The DCPR-R system was developed to capture clinically relevant psychosomatic phenomena not encompassed by conventional psychiatric classifications such as the Diagnostic and Statistical Manual of Mental Disorders (DSM) or the International Classification of Diseases (ICD). In the Italian version, the semi-structured interview has shown good inter-rater reliability (Cohen’s κ > 0.70 for most syndromes) and construct validity through associations with psychological distress, quality of life, and health outcomes [6,24]. Moreover, the revised version provides improved operational definitions and updated syndromic criteria based on recent clinical evidence.

The DCPR-R-SSI has been previously employed in a variety of clinical populations, including patients with cardiovascular diseases [7], functional gastrointestinal disorders [25], fibromyalgia [11], oncology [9,10]. Notably, it has also been applied to patients with IBD to assess the persistence of DCPR syndromes over time [12].

#### 2.3.3. Lifestyle-Related Behaviors

Lifestyle behaviors were assessed using an adapted version of the GOSPEL Questionnaire [21], a brief, structured interview developed to evaluate health-related behaviors in clinical populations. The version adopted in the present study was specifically designed for individuals with chronic medical conditions and included selected items targeting physical activity, dietary habits, alcohol consumption, and smoking, in line with the hypotheses of the current research.

Physical activity was measured through two self-report items. The first item assessed the frequency of physical exercise and was scored on a 4-point scale ranging from 0 (never or occasionally) to 3 (more than once a day). The second item evaluated the intensity of the activity performed, also using a 4-point scale from 0 (low) to 3 (very high). This dual approach allowed for the simultaneous assessment of both behavioral engagement and perceived physical exertion.

Dietary habits were evaluated using a composite dietary quality score based on participants’ reported consumption frequencies of specific food groups, namely vegetables, fruits, fish, white meat, red meat, processed meat, and dairy products. Each food category was scored according to its consistency with the dietary recommendations issued by the Italian National Research Council for Agriculture and Nutrition [26]. Higher scores reflected healthier eating patterns. In cases where participants’ responses could not be clearly categorized (e.g., due to dietary restrictions), a neutral score (0) was assigned. The total dietary score was then calculated by summing the values attributed to each food item, providing a global indicator of dietary quality, ranging from −14 to +14.

Alcohol consumption was assessed through a single item investigating the frequency of alcohol use. Responses were coded from 0 (never or occasionally) to 3 (more than once per day), allowing for a basic classification of drinking behaviors.

Finally, smoking behavior was operationalized using two indicators. The first was a binary variable indicating smoking status, with values of 0 for non-smokers and 1 for current smokers. The second indicator assessed cigarette consumption, quantified as the self-reported number of cigarettes smoked per day.

### 2.4. Data Analysis

Statistical analyses followed pre-registered protocols (OSF registration: https://osf.io/swa34 (accessed on 22 July 2025)) and were conducted with R (v4.4.3) [27] on RStudio (v2024.12.1.563) [28]. Statistical analysis scripts were generated using Claude Sonnet 4 (Anthropic) between July and August 2025 and subsequently reviewed and edited by the authors. As a preliminary step, descriptive analyses were performed on sociodemographic and clinical variables.

For the first aim, hierarchical clustering using complete linkage and Jaccard distance was performed to explore potential clustering solutions based on psychosomatic diagnoses, using R packages cluster (v2.1.8) [29], dendextend (v1.19.1) [30] and clustercrit (v1.3.0) [31].

A sensitivity analysis was conducted to assess whether the number and prevalence of psychosomatic syndromes included in the clustering procedure influenced the resulting cluster structure. Specifically, two variable sets were compared: a broader set including all syndromes with a prevalence of at least 5% in the total sample, and a narrower set including only those with a prevalence of at least 10%. For the broader set, clustering solutions with *k* ranging from 2 to 6 were tested, while for the narrower set, *k* ranged from 2 to 5. These ranges were selected based on sample size, number of variables, and the need to avoid overly small clusters.

For each selected solution, a colored dendrogram, a binary heatmap of patient profiles by cluster, and a frequency table of syndromes within clusters were generated. To evaluate clustering solutions, internal validation indices were computed—namely, the average silhouette width (*ASW*) (target ≥ 0.25) [32] and the Calinski–Harabasz index (*CH*) [33]. Additionally, 500 bootstrap replications of the gap statistic were conducted to identify the first *k* < *k*_max_ satisfying the criterion: Gap(*k*) ≥ Gap(*k* + 1) − *SE*(*k* + 1) [34]. The optimal number of clusters was selected by maximizing these indices and prioritizing solutions that met multiple criteria simultaneously. Any solution yielding clusters with fewer than 5 patients was discarded.

Cluster membership was coded as a categorical variable for all patients. Those presenting none of the psychosomatic syndromes included in the final clustering solution were assigned to cluster 0, which served as the reference category in subsequent regression analyses.

For the second aim, multiple models were fitted depending on the type of dependent variable.

To examine the relationship between psychosomatic disorders and dietary habits, as well as the number of smoked cigarettes, four separate multiple linear regression models were planned—two for each outcome. In the first set of models, psychosomatic syndromes were included as predictors, with IBD severity added as a covariate. A second set of models was planned using cluster membership instead of individual syndromes, to assess whether the clustering structure explained similar or different patterns of association. All analyses were conducted in R using specialized packages (car v3.1.3 [35], lmtest v0.9.40 [36], sandwich v3.1.1 [37,38], nortest v1.0.4 [39], lm.beta v1.7.2 [40]) for regression diagnostics and robust inference. Additionally, McDonald’s omega was calculated for the dietary habit score, to ensure reliability of the measure.

After detecting severe non-normality in residuals for the number of smoked cigarettes, the corresponding linear regression models were replaced with two zero-inflated negative binomial (ZINB) generalized linear models. These models were more appropriate for count data with overdispersion and excess zeros, and allowed modeling of smoking propensity without requiring a separate logistic component. ZINB models were fitted using additional specialized R packages (pscl v1.5.9 [41,42], MASS v7.3.64 [43], AER v1.2.15 [44]) for model estimation, diagnostic testing, and model comparison.

To examine the relationship between psychosomatic disorders and physical activity (both intensity and frequency), as well as alcohol consumption, three separate ordered probit models were fitted—one for each outcome variable, using additional R packages (brant v0.3.0 [45]) for model estimation and assumption testing. Each model was fitted twice: once with individual syndromes as predictors and once with cluster membership as predictors, with IBD severity added as a covariate in all models. For these models, if the proportional odds assumption was violated, partial proportional odds models or multinomial logistic regression were employed using additional R packages (VGAM v1.1.13 [46], nnet v7.3.20 [43]) to accommodate varying effects across response categories.

Outlier evaluation and handling were conducted for all analyses using pre-registered criteria.

For linear regressions, any records with high influence (Cook’s distance > 4/*N*), high standardized residuals (|Std. Residuals| ≥ 2.5), and/or high leverage (leverage > 2(*k* + 1)/*N*, where *k* = number of predictors and *N* = sample size) were considered as outliers.

For ZINB regressions, any records with high Pearson residuals (|Pearson Residuals| ≥ 2.5), high deviance residuals (|Deviance Residuals| ≥ 2.5), and/or extreme prediction differences (absolute difference between observed and predicted values > 95th percentile of the distribution) were considered as outliers.

For ordered probit models, any records with high Cook’s distance (>4/*N*), high standardized residuals (|Std. Residuals| ≥ 2.5), and/or extreme prediction differences (≥2 ordinal levels between observed and predicted categories) were considered as outliers.

Outliers were handled following a conservative approach prioritizing sample representativeness and data accuracy. All identified outliers were verified for correctness and retained when they represented legitimate clinical observations that did not significantly compromise model stability. For the clustering analysis, one observation that substantially interfered with cluster structure was excluded after verification. Clustering outliers were reassigned when possible to the cluster with minimum centroid distance, provided this improved their silhouette score without reducing the overall solution quality.

Model assumptions were checked for each analysis, and appropriate remedies were applied when necessary.

A two-tailed *p*-value < 0.05 was considered statistically significant. Effect sizes with confidence intervals were reported alongside significance tests. To account for multiple comparisons across regression models within each health behavior domain (dietary habits, smoking, physical activity, and alcohol consumption), *p*-values were adjusted using the Benjamini–Hochberg False Discovery Rate (FDR) procedure.

#### Exploratory Analyses

Three exploratory analyses were conducted to examine: differences in psychosomatic profiles between UC and CD, the influence of psychopharmacological treatment on the observed associations, and the association between the number of DCPR syndromes per patient and health behaviors.

For the first exploratory analysis, between-group differences were assessed using chi-square tests when assumptions were met (i.e., at least 80% of cells with expected frequencies ≥ 5), or Fisher’s exact test otherwise. For single syndrome comparisons, Fisher’s exact test computed exact *p*-values for 2 × 2 contingency tables. For cluster distribution (6 × 2 table), when Fisher’s exact test was required, *p*-values were approximated through Monte Carlo simulation with 10,000 replications due to computational constraints. To identify which specific clusters contributed most to any overall difference between UC and CD, Pearson’s standardized residuals were examined from the chi-square model, with absolute values > 2 indicating substantial deviations from expected frequencies

For the second analysis, all described regression models were replicated with antidepressant and anxiolytic use as covariates.

Finally, for the third analysis, b, all described regression models were additionally fitted with the total number of psychosomatic syndromes as single predictor and IBD severity as covariate. These analyses were conducted post hoc and were not pre-registered in the original study protocol.

## 3. Results

### 3.1. Sample Characteristics

The final sample comprised 111 adult patients with a confirmed diagnosis of IBD (CD = 40, UC = 71), all of whom were in a clinically defined phase of low disease activity at the time of assessment. Participants had a median age of 49 years (*IQR* = 21.5) and a median disease duration of 9 years (*IQR* = 12).

Sociodemographic characteristics of the sample are reported in Table 1.

The distributions of clinical variables of the sample are reported in Table 2.

As for lifestyle behaviors, the number of daily cigarettes among smokers was not normally distributed (*W* = 0.874, *p* = 0.002), with a median of 7 (*IQR* = 6). The composite dietary habits score presented an average value of 2.77 (*SD* = 3.79), indicating a weak trend towards positive values on the scale. Health behaviors characteristics of the sample are reported in Table 3.

The distribution of DCPR syndromes in the sample, both overall and by IBD type, is reported in Table 4.

### 3.2. Clustering Sensitivity Analysis

Hierarchical clustering analysis was conducted on a subset of 76 patients (68.4% of the total sample) who exhibited at least one of the six psychosomatic syndromes selected for clustering. Initial clustering solutions (*k* = 2:6) failed to meet minimum validation criteria. Following the pre-registered protocol, outlier analysis was conducted across all clustering solutions to identify persistent outliers interfering with identification of optimal solutions. Outlier analysis revealed 32 outliers across the five solutions, with seven patients demonstrating persistent outlier behavior (present in ≥ 3 solutions). An iterative approach was employed, with the most persistent outliers being individually removed to evaluate potential improvements. Removal of patient ID 15, who exhibited the most consistent outlier pattern, resulted in substantial improvements to validation indices and enabled generation of viable clustering solutions for *k* = 3:6. Additional outlier removal did not yield significant improvements and was avoided to maintain sample representativeness, resulting in a final analytical sample of 75 patients.

**Figure 1:**
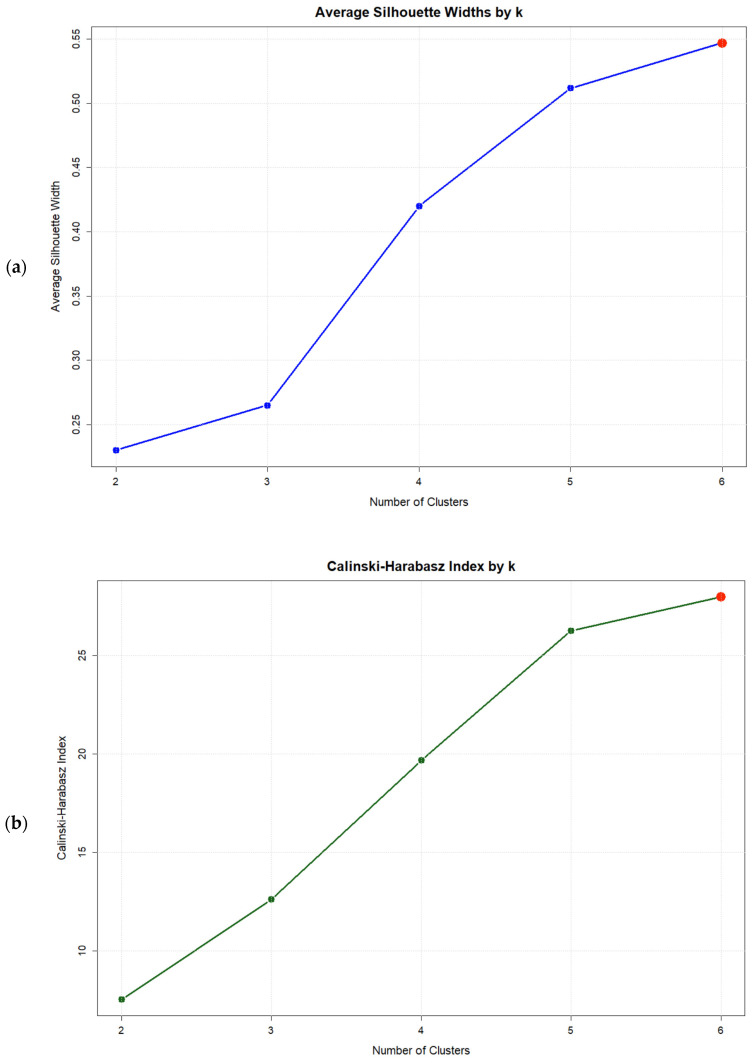

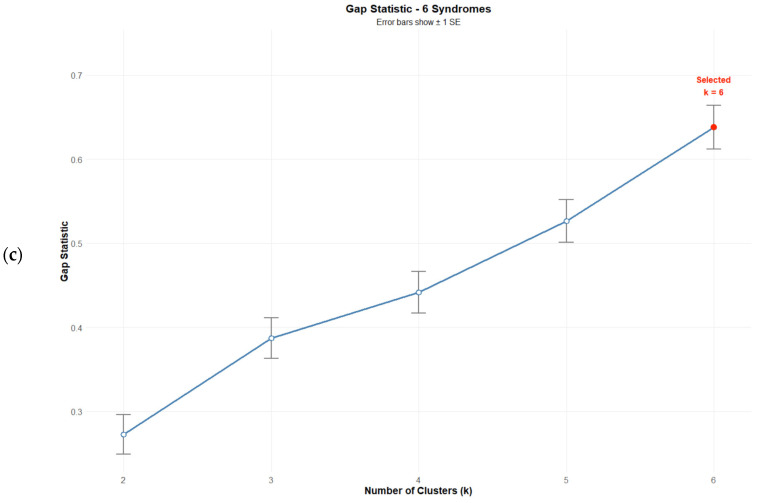
Validation index plots for hierarchical clustering solutions: (**a**) Average silhouette width, (**b**) Calinski–Harabasz index, and (**c**) Gap statistic for *k* = 2:6 Solutions based on six psychosomatic syndromes (*n* = 75). Red dots highlight the selected solution.

Evaluation of clustering solutions revealed that while no solution with *k* < *k*_max_ satisfied the gap criterion [Gap(*k*) ≥ Gap(*k* + 1) − *SE*(*k* + 1)], the *k* = 6 solution achieved the highest gap statistic value (0.527) (Figure 1c), representing the optimal solution within the tested range. This solution also maximized both the Average Silhouette Width (0.547) and the Calinski–Harabasz index (27.99) (Figure 1a,b). Validation indices for all solutions are presented in Table 5.

For the *k* = 6 solution, four specific outliers were initially identified. Following the reassignment protocol, an additional outlier emerged, resulting in five outliers being reassigned to their nearest cluster centroids. This process significantly improved solution statistics (*ASW* = 0.58, *CH* = 30.83, Gap = 0.638). The final distribution of patients and psychosomatic syndromes across the six clusters is presented in Table 6.

Following exclusion of persistent somatization, hierarchical clustering analysis was conducted on the remaining five syndromes across 75 subjects. This analysis yielded clustering solutions ranging from *k* = 2:5, with only the *k* = 4 and *k* = 5 solutions meeting the minimum validation criteria.

**Figure 2:**
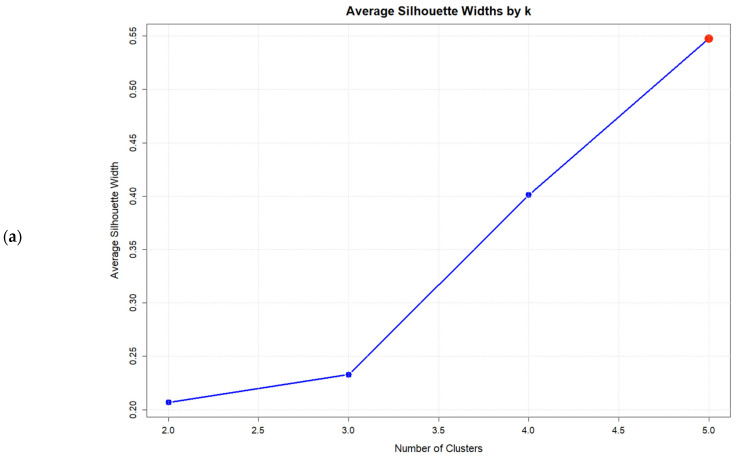

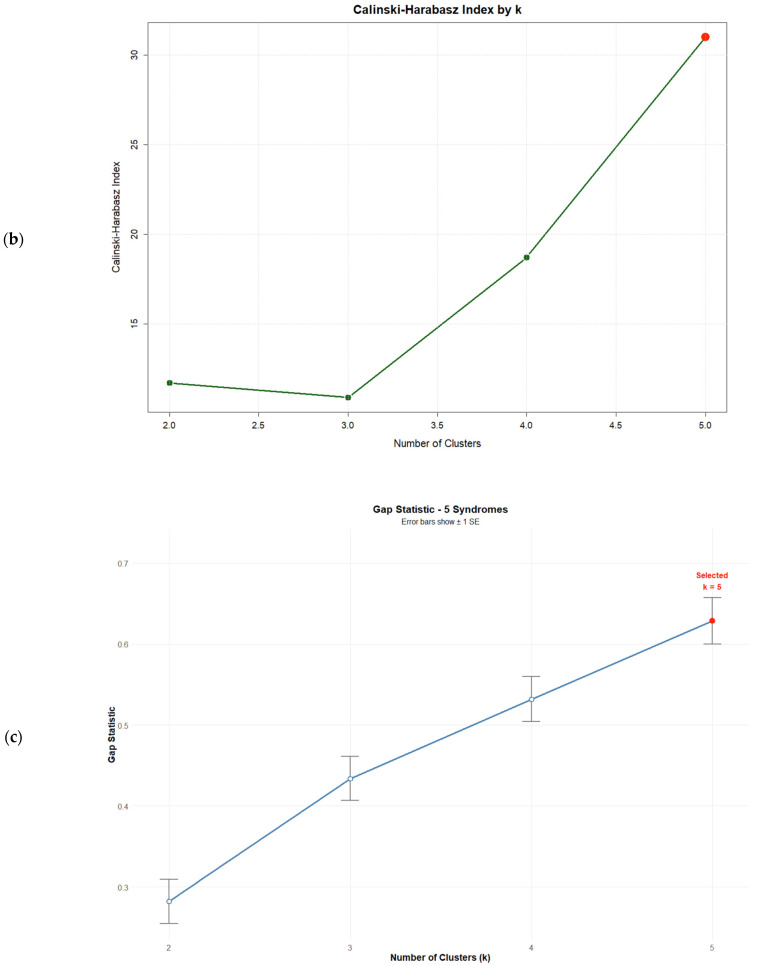
Validation index plots for hierarchical clustering solutions: (**a**) Average silhouette width, (**b**) Calinski–Harabasz index, and (**c**) Gap statistic for *k* = 2:5 solutions based on five psychosomatic syndromes (*n* = 75). Red dots highlight the selected solution.

Consistent with the six-syndrome analysis, no solution with *k* < *k*_max_ satisfied the gap criterion. The *k* = 5 solution achieved the highest validation indices, maximizing the Average Silhouette Width (0.548), the Calinski–Harabasz index (31.01), and the Gap statistic (0.532), thereby representing the optimal solution within the tested range (Figure 2). Validation indices for all solutions are presented in Table 7.

For the *k* = 5 solution, five specific outliers were initially identified. Following the reassignment protocol, all five outliers were reassigned to their nearest cluster centroids. This process significantly improved solution statistics (*ASW* = 0.564, *CH* = 34.43, Gap = 0.629). The final distribution of patients and psychosomatic syndromes across the five clusters is presented in Table 8.

Comparison between the two clustering solutions revealed substantial correspondence in psychosomatic profiles:

- Cluster 1 maintained similar profiles in both analyses, featuring high prevalence of irritable mood and type A behavior;
- Alexithymia-dominant profiles were represented by cluster 3 (6-syndrome solution) and cluster 2 (5-syndrome solution);
- Demoralization combined with allostatic overload appeared as cluster 5 (6-syndrome) versus cluster 4 (5-syndrome);
- Type A behavior associated with alexithymia characterized cluster 6 (6-syndrome) versus cluster 5 (5-syndrome);
- Partial correspondence was observed between cluster 4 of the 6-syndrome solution (allostatic overload and type A behavior with demoralization) and Cluster 3 of the 5-syndrome solution (allostatic Overload with type A Behavior).

Notably, cluster 2 of the 6-syndrome solution, characterized by high prevalence of Allostatic Overload alone, had no equivalent in the 5-syndrome clustering.

Given the minimal differences between validation indices for both solutions (Δ*ASW* = 0.016, Δ*CH* = 3.6, ΔGap = 0.007), the *k* = 6 solution was selected for subsequent analyses. This decision was based on the potential clinical relevance of the additional allostatic overload cluster for patient stratification. Visual representations of the *k* = 5 solution from the 5-syndrome analysis are provided in the Supplementary Materials (Figures S1 and S2).

**Figure 3:**
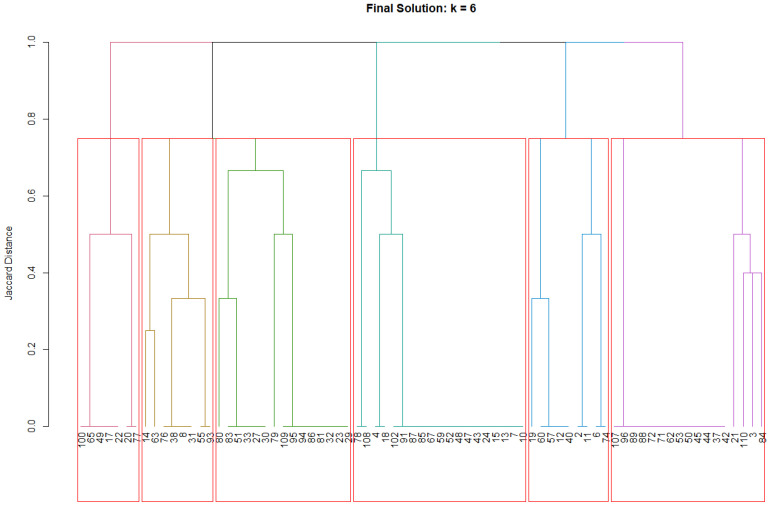
Colored dendrogram for solution *k* = 6. Colored branches represent the six identified clusters, with each color corresponding to a distinct patient cluster. The horizontal red line indicates the cut height used to obtain the final six-cluster solution.

**Figure 4:**
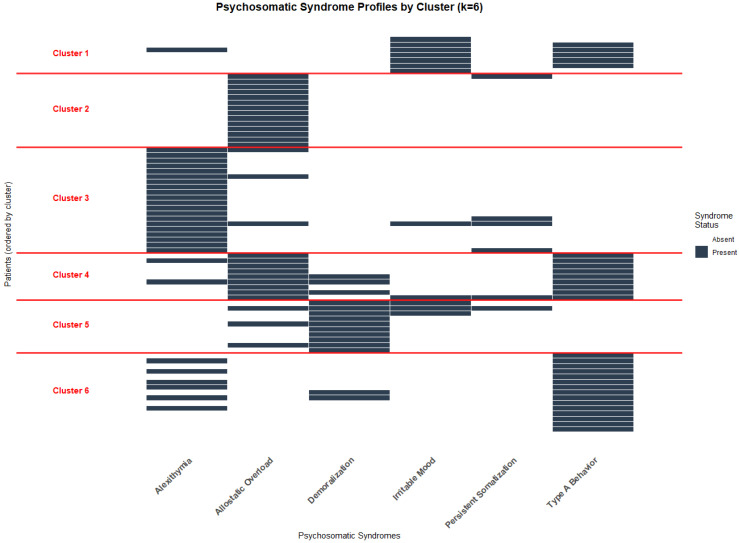
Binary heatmap for solution *k* = 6.

The final *k* = 6 clustering solution identified six distinct psychosomatic profiles with cluster sizes ranging from 7 to 20 patients (9.3–26.7% of the sample). Each cluster demonstrated distinct syndrome patterns with internal consistency ranging from acceptable to excellent (individual cluster silhouette widths: 0.397–0.890, all >0.350) and clear between-cluster differentiation, as evidenced by the binary heatmap patterns. Visual representations of the clustering structure are provided in Figure 3 and Figure 4. The colored dendrogram (Figure 3) illustrates the hierarchical structure and optimal cutting point for *k* = 6, while the binary heatmap (Figure 4) visualizes the distinct psychosomatic profiles that characterize each cluster. These visual analyses confirm both the clinical interpretability and statistical robustness of the clustering solution.

The six clusters demonstrated distinct psychosomatic profiles:

- Cluster 1 (*n* = 7, 9.3%) was characterized primarily by irritable mood (100%) and type A behavior (75%), and was designated “Irritability”;
- Cluster 2 (*n* = 14, 18.7%) featured exclusively allostatic overload (100%) and was designated “Pure allostatic overload”;
- Cluster 3 (*n* = 20, 26.7%) was dominated by alexithymia (100%) and was designated “Pure Alexithymia”;
- Cluster 4 (*n* = 9, 12%), characterized by universal presence of type A behavior (100%) and allostatic overload (100%), also exhibited demoralization in one-third of patients (33.3%). This profile was designated “Overwhelmed Type A Behavior”;
- Cluster 5 (*n* = 10, 13.3%) showed universal demoralization (100%) with moderate co-occurrence of allostatic overload (30%) and irritable mood (30%). Given this symptom constellation, it was designated “Subclinical Depressive Mood”;
- Cluster 6 (*n* = 20, 26.7%) was characterized by universal type A behavior (100%) and moderate co-occurrence of alexithymia (40%), and was designated “Alexithymic Type A Behavior”.

Detailed characterization of the clustering solution is presented in Table 6.

### 3.3. Dietary Habits

Associations between psychosomatic syndromes and dietary habits were examined using multiple linear regression models with two complementary approaches: individual syndromes as predictors and cluster membership as predictors, both including IBD severity as covariate. McDonald’s Omega for the composite dietary score was 0.347, indicating poor internal consistency reliability for this measure.

#### 3.3.1. Individual Syndromes Model

Assumption testing revealed potential residual autocorrelation (Breusch-Godfrey test *p* = 0.037; Durbin-Watson = 2.36). To address this issue while preserving coefficient interpretation, Heteroskedasticity and Autocorrelation Consistent (HAC) standard errors were employed using the Newey-West estimator [47]. This approach was preferred over structural correction methods (e.g., Prais-Winsten) because it maintains original coefficient estimates while providing robust inference. All other model assumptions were satisfied, and outlier assessment confirmed data integrity (*N* = 111).

The regression model failed to achieve statistical significance, *F*(7, 103) = 0.038, *p* = 0.999, explaining only 0.26% of dietary score variance (*R*^2^ = 0.0026). Accordingly, no individual syndrome significantly predicted dietary quality.

#### 3.3.2. Cluster-Based Model

All model assumptions were satisfied and outlier assessment confirmed data integrity (*n* = 110). This model also failed to achieve statistical significance, *F*(7, 102) = 0.36, *p* = 0.925, explaining 2.4% of variance (*R*^2^ = 0.024). No psychosomatic cluster significantly differed from the reference group regarding dietary quality.

### 3.4. Smoking Habits

Association between psychosomatic syndromes and smoking behaviors were examined using zero-inflated negative binomial regression with two complementary approaches. The first model tested individual syndrome effects, while the second evaluated cluster membership effects. Both approaches examined predictors for smoking propensity and cigarette consumption intensity among smokers, with IBD severity included as a covariate.

#### 3.4.1. Individual Syndromes Model

The ZINB model converged successfully (*N* = 111, with 73% structural zeros) and all model assumptions were satisfied. The dispersion parameter θ = 3.31 (*SE* = 0.42) confirmed substantial overdispersion, supporting the negative binomial specification. Outlier assessment confirmed data integrity.

Analysis revealed no significant associations between individual psychosomatic syndromes and smoking behaviors. In the count component (modeling cigarette quantity among smokers), no syndrome reached statistical significance (all *p* > 0.05). In the zero-inflation component (modeling probability of being a non-smoker), no individual syndrome demonstrated significant associations (all *p* > 0.05).

#### 3.4.2. Cluster-Based Model

The ZINB model converged successfully (*n* = 110, with 72.7% structural zeros), and all model assumptions were satisfied. The dispersion parameter θ = 3.09 (*SE* = 0.42) confirmed substantial overdispersion, supporting the negative binomial specification. Outlier assessment confirmed data integrity.

In the count component (modeling cigarette quantity among smokers), no cluster membership achieved statistical significance (all *p* > 0.05). In the zero-inflation component (modeling probability of being a never-smoker), membership in the Pure Alexithymia cluster was associated with reduced probability of being a never-smoker (*OR* = 0.26, 95% *CI*: 0.07–0.90, *p* = 0.033), corresponding to a 3.85-fold increase in smoking odds. However, this association lost statistical significance after Benjamini–Hochberg correction (*p* = 0.400). Coefficients for the zero-inflation component are presented in Table 9.

### 3.5. Physical Activity

Associations between psychosomatic syndromes and physical activity frequency were assessed using two separate ordered probit models: one with individual syndromes as predictors and another with cluster membership as predictors, both including IBD severity as a covariate. The same modeling approach was applied to examine associations between psychosomatic syndromes and physical activity intensity.

#### 3.5.1. Individual Syndromes Model for Physical Activity Frequency

The proportional odds assumption was evaluated using the Brant test, which indicated a violation (*p* < 0.05). Following the pre-registered protocol, a partial proportional odds model was attempted to accommodate this violation, but this failed to converge due to quasi-separation arising from sparse data (one cell with *n* = 0 in the IBD severity × outcome contingency table). As the pre-specified alternative, a multinomial logistic regression model was fitted and compared to the ordered probit using a likelihood ratio test. The test showed no significant difference between models (χ^2^ = 20.761, *df* = 14, *p* = 0.108), indicating that the proportional odds violation did not meaningfully compromise model fit or interpretation. Therefore, the more parsimonious ordered probit model was retained, acknowledging that while the proportional odds assumption was technically violated, this violation did not substantially impact the validity of the conclusions. All other model assumptions were satisfied, and outlier assessment confirmed data integrity.

The ordered probit model converged successfully (*n* = 111). The overall model showed limited explanatory power (McFadden pseudo-*R*^2^ = 0.033) No individual psychosomatic syndrome demonstrated statistically significant associations with physical activity frequency (all *p* > 0.05). The IBD severity covariate was also non-significant (*p* > 0.05).

#### 3.5.2. Cluster-Based Model for Physical Activity Frequency

The proportional odds assumption was evaluated using the Brant test which indicated a violation (*p* < 0.05). However, further model comparisons suggested that this violation was not substantively impactful. A partial proportional odds (PPO) model was successfully fitted to accommodate the detected violation, but a likelihood ratio test comparing PPO to the standard ordered probit model yielded no improvement in model fit (*LR* = 0, *p* = 1.0). This suggests that the PPO model, while technically more flexible, did not offer meaningful advantages in terms of explanatory power or interpretability.

A multinomial logistic regression model was also fitted and compared to the ordered probit model. Although the likelihood ratio test indicated a statistically significant improvement in fit (*LR* = 30.23, *df* = 14, *p* = 0.007), the multinomial model exhibited numerical instability due to quasi-separation and sparse data, including four empty cells in cluster-outcome contingency table and two empty cells in the IBD severity-outcome contingency table. These issues undermine the reliability of the parameter estimates and limit the model’s practical applicability.

In line with the pre-registered analysis plan and given that the PPO model did not outperform the ordered probit and the multinomial model raised robustness concerns, the ordered probit model was retained. The proportional odds violation was acknowledged but considered non-substantive, as it did not materially affect model convergence, interpretability, or conclusion validity. All other model assumptions were satisfied, and outlier assessment confirmed data integrity.

The obtained probit model converged successfully (*n* = 110) with limited explanatory power (McFadden pseudo-*R*^2^ = 0.037). Membership in the Overwhelmed Type A Behavior cluster was significantly associated with reduced odds of higher physical activity frequency (*OR* = 0.27, 95% *CI*: 0.09–0.80, *p* = 0.018) compared to the reference group (no cluster membership). However, this association lost statistical significance after Benjamini–Hochberg correction (*p* = 0.434). No other predictors demonstrated significant associations with physical activity frequency. Coefficients for all predictors are presented in Table 10.

#### 3.5.3. Individual Syndromes Model for Physical Activity Intensity

The proportional odds assumption was evaluated using the Brant test, which indicated a violation related to the irritable mood predictor (*p* < 0.05). A partial proportional odds model was attempted to accommodate this violation, but this failed to converge. As the pre-specified alternative, a multinomial logistic regression model was fitted and compared to the ordered probit using a likelihood ratio test. The test showed no significant difference between models (χ^2^ = 17.145, *df* = 14, *p* = 0.249), indicating that the proportional odds violation did not meaningfully compromise model fit or interpretation. Therefore, consistent with previous analyses, the more parsimonious ordered probit model was retained. All other model assumptions were satisfied, and outlier assessment confirmed data integrity.

The ordered probit model converged successfully (*N* = 111) with limited explanatory power (McFadden pseudo-*R*^2^ = 0.026). No individual psychosomatic syndromes demonstrated statistically significant associations with physical activity intensity (all *p* > 0.05). The IBD severity covariate was also non-significant (*p* > 0.05).

#### 3.5.4. Cluster-Based Model for Physical Activity Intensity

The ordered probit model converged successfully (*n* = 110), and all model assumption were satisfied. The overall model showed limited explanatory power (McFadden pseudo-*R*^2^ = 0.019). No cluster membership demonstrated statistically significant associations with physical activity intensity (all *p* > 0.05). The IBD severity covariate was also non-significant (*p* > 0.05).

### 3.6. Alcohol Consumption

Associations between psychosomatic syndromes and frequency of alcohol consumption were assessed using two separate ordered probit models: one with individual syndromes as predictors and another with cluster membership as predictors, both including IBD severity as a covariate.

#### 3.6.1. Individual Syndromes Model

The ordered probit model converged successfully (*N* = 111) and all model assumptions were satisfied. The overall model showed limited explanatory power (McFadden pseudo-*R*^2^ = 0.064). Irritable mood was associated with lower frequency of alcohol consumption (*OR* = 0.29, 95% *CI*: 0.09–0.92, *p* = 0.035), while type A behavior was associated with higher consumption frequency (*OR* = 1.87, 95% *CI*: 1.06–3.29, *p* = 0.030). However, both associations lost statistical significance after Benjamini–Hochberg correction (adjusted *p* = 0.210). No other predictors demonstrated significant associations with alcohol consumption frequency. Coefficients for all predictors are presented in Table 11.

#### 3.6.2. Cluster-Based Model

The ordered probit model converged successfully (*n* = 110) and all model assumptions were satisfied. The overall model showed limited explanatory power (McFadden pseudo-*R*^2^ = 0.027). No cluster membership demonstrated statistically significant associations with alcohol consumption frequency (all *p* > 0.05). The IBD severity covariate was also non-significant (*p* > 0.05).

### 3.7. Exploratory Analyses

First, associations between IBD type (UC vs. CD) and psychosomatic syndromes and clusters were assessed descriptively and statistically tested using chi-square tests and, when needed, Fisher’s exact test. Second, all regression models were replicated with antidepressant and anxiolytic use as additional covariates. Finally, associations between the number of DCPR syndromes per patient and health behaviors were assessed exploratively by replicating all previous models with the number of DCPR syndromes as a predictor and IBD severity as a covariate.

#### 3.7.1. Psychosomatic Profiles of UC and CD Patients

The distribution of psychosomatic syndromes and cluster membership across UC and CD patients is reported in Table 4 and Table 12, respectively, with visual representations provided in Supplementary Figure S3.

The chi-square test revealed no significant association between the presence of any DCPR syndrome and disease type (χ^2^ = 3.32, *p* = 0.069). Similarly, individual syndrome comparisons showed no significant differences between UC and CD patients. Four chi-square tests were conducted for type A behavior (χ^2^ = 1.76, *p* = 0.184), demoralization (χ^2^ = 0.00, *p* = 1.00), alexithymia (χ^2^ = 0.18, *p* = 0.669), and allostatic overload (χ^2^ = 0.22, *p* = 0.637), while Fisher’s exact tests were used for persistent somatization (*p* = 0.701) and irritable mood (*p* = 1.00) due to low expected cell frequencies.

Regarding cluster distribution, Fisher’s exact test with Monte Carlo simulation (10,000 replications) was employed due to violation of chi-square assumptions (only 50% of cells with expected frequencies ≥ 5). The test showed no significant difference in cluster membership between UC and CD patients (*p* = 0.565). Consistent with this finding, examination of Pearson’s standardized residuals revealed no values exceeding |2|, indicating no specific cluster showed substantial over- or under-representation in either disease group.

#### 3.7.2. Models Replication with Additional Covariates

The introduction of anxiolytic and antidepressant use as covariates in all models did not lead to substantial changes in the results, with one notable exception. In the ZINB model assessing the association between cluster membership and smoking status, the addition of these covariates revealed that patients taking anxiolytics had significantly lower odds of being non-smokers (*OR* = 0.12, 95% *CI*: 0.02–0.87, *p* = 0.036). Concurrently, membership in the Pure Alexithymia cluster showed a slightly stronger association with smoking status when adjusting for psychopharmacological treatment (*OR* = 0.22, 95% *CI*: 0.06–0.81, *p* = 0.023) compared to the original model (*OR* = 0.26, 95% *CI*: 0.07–0.90, *p* = 0.033).

#### 3.7.3. Association Between Number of DCPR Syndromes and Dietary Habits

Assumption testing revealed potential residual autocorrelation (Breusch-Godfrey test *p* = 0.041; Durbin-Watson = 2.35). To address this issue while preserving coefficient interpretation, HAC standard errors were employed using the Newey-West estimator [47] as in previous analyses. All other model assumptions were satisfied, and outlier assessment confirmed data integrity.

The regression model failed to achieve statistical significance, *F*(2, 108) = 0.002, *p* = 0.998, explaining virtually none of the dietary score variance (*R*^2^ = 0.00004). Accordingly, the number of DCPR syndromes was not a significant predictor of dietary quality (*p* = 0.982).

#### 3.7.4. Association Between Number of DCPR Syndromes and Smoking Habits

The ZINB model converged successfully (*N* = 111, with 73% structural zeros) and all model assumptions were satisfied. The dispersion parameter θ = 3.07 (*SE* = 0.43) confirmed substantial overdispersion, supporting the negative binomial specification. Outlier assessment confirmed data integrity.

Analysis revealed no significant associations between the number of psychosomatic syndromes and smoking behaviors. In the count component (modeling cigarette quantity among smokers), statistical significance was not achieved (*p* = 0.357). In the zero-inflation component (modeling probability of being a non-smoker), no significant associations with syndrome count were observed (*p* = 0.364).

#### 3.7.5. Association Between Number of DCPR Syndromes and Physical Activity

For physical activity frequency, Brant test indicated proportional odds assumption violation. Similar model comparison procedures were conducted as for the cluster-based model: the PPO model showed no improvement over the standard ordered probit (*LR* = 0, *p* = 1.0), and the multinomial logistic regression exhibited numerical instability due to quasi-separation and sparse data. Following the same rationale, the ordered probit model was retained as the primary analytic approach, acknowledging the non-substantive proportional odds violation that did not compromise model validity or interpretability. All other model assumptions were satisfied and outlier assessment confirmed data integrity. The ordered probit model converged successfully (*N* = 111) with limited explanatory power (McFadden pseudo-*R*^2^ = 0.008). The number of DCPR syndromes demonstrated no statistically significant association with physical activity frequency (*p* = 0.205).

For physical activity intensity, the ordered probit model converged successfully (*N* = 111). All model assumption were satisfied and outlier assessment confirmed data integrity. The overall model showed limited explanatory power (McFadden pseudo-*R*^2^ = 0.001), and the number of DCPR syndromes demonstrated no statistically significant association with physical activity intensity (*p* = 0.649).

#### 3.7.6. Association Between Number of DCPR Syndromes and Alcohol Consumption

For alcohol consumption frequency, the ordered probit model converged successfully (*N* = 111). All model assumptions were satisfied, and outlier assessment confirmed data integrity. The overall model showed limited explanatory power (McFadden pseudo-R^2^ = 0.002) and the number of DCPR syndromes demonstrated no statistically significant association with alcohol consumption frequency (*p* = 0.871).

## 4. Discussion

The aim of the present study was to identify the most common psychosomatic profiles of IBD patients with low disease activity and examine their association with health behaviors. Distinct psychosomatic syndrome clusters were identified, and several potential associations with lifestyle factors (smoking, physical activity, alcohol consumption) emerged, although these findings require replication in larger samples. In the following sections, results and their implications for clinical practice and research are discussed.

### 4.1. Prevalence of Psychosomatic Syndromes

Regarding psychosomatic syndromes, descriptive analyses revealed the presence of at least one psychosomatic syndrome in 69.4% of the sample (*n* = 77), with 10.8% of patients (*n* = 12) presenting at least 3 syndromes at the same time. These findings assume particular relevance when considering that these syndromes carry a significant clinical burden [48], are often underdiagnosed and undertreated in clinical settings compared to classic DSM syndromes [48,49], and demonstrate important persistence over time. A recent study by Gostoli et al. [12], conducted on a subset of the present study sample, found that 43% of all psychosomatic syndromes observed in IBD patients were maintained at a four-year follow-up.

Seventy-six and one-tenth percent of UC and 57.5% of CD patients presented at least one DCPR syndrome. However, post hoc exploratory analyses did not identify this difference as statistically significant. Furthermore, post hoc exploratory comparisons of individual syndrome prevalence between these two groups revealed no significant differences. These findings should be interpreted with caution given their exploratory nature and the limited statistical power to detect small effects.

In the overall sample, the most prevalent psychosomatic syndromes in the sample were alexithymia (26.1%), type A behavior (26.1%), allostatic overload (26.1%), demoralization (13.5%), irritable mood (10.8%) and persistent somatization (6.3%). In the same study by Gostoli et al. [12], these syndromes showed persistence rates ranging from 20% (irritable mood) to 72% (allostatic overload) over four years.

Regarding alexithymia, numerous studies have investigated the relationship between this psychosomatic syndrome and gastrointestinal disorders. A review by Carrozzino & Porcelli [50] reported alexithymia prevalence in IBD patients similar to the one in the present sample (30–35%), which was higher than in the general population yet lower than in functional gastrointestinal disorders (FGID) (66–87%). However, as noted in the same review, other studies reported lower alexithymia prevalence in IBD, sometimes similar to or even lower than general population rates. This appears consistent with another review by Martino et al. [51] conducted on more recent articles. Although consensus regarding the effective prevalence of alexithymia in IBD patients remains to be established, the presence of this psychosomatic syndrome may be associated with physiological processes that could lead to increased inflammatory activities [52]. Furthermore, alexithymia may be related to somatosensory amplification, resulting in lower threshold for pain and symptom perception [52]. Finally, in a study by Porcelli et al. [25], alexithymia had a significantly higher presence in FGID patients not responding to therapy at a six-month follow-up compared to responsive patients.

Regarding type A behavior, some evidence suggests associations between type A behavior and gastrointestinal conditions [53,54,55,56,57], but the evidence is limited and outdated, requiring cautious interpretation. Compared to other gastrointestinal conditions, the type A behavior prevalence in this study (26.1%) exceeds the one in patients undergoing colorectal cancer screening (13.3%) and in those subsequently diagnosed with adenoma (14.3%), although type A behavior was among the most prevalent psychosomatic syndromes in both cases [58]. Similarly, type A behavior appear notably less prevalent in FGID patients (8.4%) [59]. Future studies should further evaluate the potential specific relationship between this psychosomatic syndrome and IBDs.

Research on allostatic overload in gastrointestinal disorders remains limited. Nevertheless, the prevalence reported in this study (26.1%) is comparable to that found by Gostoli et al. [58] in patients undergoing colorectal cancer screening (18%), with allostatic overload being among the most prevalent psychosomatic syndromes in these types of patients. Notably, a recent study by Zhao et al. [60] observed that higher levels of allostatic loads were associated with increased risk of developing IBD. Furthermore, in IBD patients, elevated allostatic load levels were associated with higher all-cause mortality and increased risk of complications and related surgery [58].

Regarding demoralization, few studies have investigated its presence in gastrointestinal diseases. Generally, demoralization has a prevalence of 30% in medical settings [61], which is significantly higher than the value found the in the general population (3%) [62] and compared to present sample (13.5%). However, patients undergoing colorectal cancer screening showed demoralization prevalence similar to the one in the present study (11.3%), maintained in patients subsequently diagnosed with adenomas (10.7%) [58]. Demoralization is also among the most prevalent DCPR syndromes in FGID, although the prevalence is higher than the one in the present sample (22.6%) [59].

Regarding irritable mood, the prevalence in this study (10.8%) was similar to the one found in FGID patients (10%) [59,63], while it was considerably lower in patients undergoing colorectal cancer screening (2.7%) [58]. Notably, a recent study by Luo et al. [64] proposed a bidirectional causal relationship between irritable mood and irritable bowel syndrome (IBS). Furthermore, gastrointestinal patients appear more likely to present irritable depressive symptoms compared to healthy populations, according to Kovács et al. [65]. Given these considerations, future studies should further characterize the role of irritable mood in IBD, particularly considering its close association with type A behavior [7,66], which demonstrated high prevalence in this sample.

A notable difference was observed between the prevalence of persistent somatization in the present sample (6.3%) and the one in FGID patients (33.7%) [59]. This difference may be related to the distinct symptom characteristics between these conditions. IBD activity have shown limited responsiveness to psychotherapy [67], while improvement has been associated with psychopharmacological therapy [68], suggesting that biological factors may play a more prominent role in this condition. Notably, the literature reports higher somatization levels in IBD patients with more severe symptoms [69] or higher disease activity, eventually reaching somatization levels similar to those in IBS patients [70]. Therefore, the low somatization level in this sample may be attributed to the participants’ low disease activity status.

### 4.2. Psychosomatic Profiles

Cluster analysis of patients based on six psychosomatic syndromes resulted in six distinct profiles: irritability, pure allostatic overload, pure alexithymia, overwhelmed type A behavior, subclinical depressive mood, and alexithymic type A behavior. Comparison with the five-cluster solution (excluding persistent somatization) revealed minimal statistical differences, but the inclusion of the additional “pure allostatic overload” cluster provided important clinical value, ultimately supporting selection of the six-cluster solution.

Although some differences were observed at the descriptive level in cluster membership between UC and CD patients (Table 12, Supplementary Figure S3), post hoc exploratory analyses did not reveal any significant differences between the two IBD subtypes (*p* > 0.05).

Several identified clusters demonstrated notable correspondence with previous DCPR-R syndrome classifications, underscoring the robustness of these findings. For example, the “alexithymic type A behavior” cluster corresponds precisely to the personality clinical domain to which these syndromes were assigned in the original DCPR revision [6]. This suggests that patients in this cluster may have fundamentally different approaches to illness and distinctive perspectives on themselves and other healthcare actors involved in their care [6].

Similarly, in the original DCPR framework, allostatic overload was assigned to a single domain—stress—paralleling the “pure allostatic overload” cluster identified in the present study. This could imply that patients in this cluster may exhibit psychological distress characteristics primarily associated with elevated stress levels, potentially linked to previous stressful life experiences [6].

Finally, in the original DCPR framework, a domain characterized by subclinical psychological manifestations was established, including demoralization and irritable mood. This domain shows similarities to the “subclinical depressive mood” cluster, where these subclinical manifestations are complemented by the somatic component of allostatic overload [6]. The identification of a cluster with these characteristics suggests that some IBD patients may present subclinical features that could cause psychological distress while simultaneously being underrecognized by clinicians who focus on threshold criteria for treatment.

Regarding the “Irritability” cluster, numerous previous studies have demonstrated consistent associations between irritable mood and type A behavior across multiple clinical populations [7,71], including other cluster analysis attempts [49,72], ultimately leading to their formal grouping in the “Irritability” DCPR cluster [73]. As this cluster is more commonly associated with cardiac diseases [7,71], patients in this cluster should be evaluated for the potential presence or development of cardiovascular disease, although evidence characterizing irritability as a risk factor for cardiovascular disease development remains inconclusive [74,75,76,77] and should therefore be interpreted cautiously. Future studies should investigate the potential specific role of irritability in IBD.

The “pure alexithymia” cluster was previously identified in a study of 188 alexithymic patients from diverse medical settings, where 29.3% of cases presented without psychiatric comorbidities and with limited associated psychosomatic syndromes [78]. The identification of a cluster comprising patients with this condition alone may be related to the relatively high prevalence of alexithymia in IBD patients, as reported in previous studies [50] and confirmed in the present one. Notably, in literature alexithymia is associated with more intense somatic symptom manifestation, primarily due to enhanced somatosensory perception and associated increased pain sensitivity [50]. Since somatization has been linked to disease activity in IBD [69,70], patients in this cluster may exhibit and report heightened physical and somatic symptoms during disease activity flares. Preventive interventions for these patients, such as Dialectical Behavior Therapy (DBT) [79] or mindfulness-based interventions [80], could provide protective effects against somatization and pain perception during disease exacerbations.

The “overwhelmed type A behavior” cluster, to our knowledge, has not been previously observed in studies of DCPR syndromes. A potential explanation for the co-occurrence of these syndromes may be found in the association between Type A behavior and burnout syndrome, although evidence on this relationship remains ambiguous [81,82,83,84]. Since burnout encompasses components related to allostatic overload and demoralization [85], the clustering of these three syndromes in affected patients may represent a subclinical manifestation of burnout. Given that studies have demonstrated work-related difficulties and productivity loss in IBD patients [86,87], future research should further characterize this cluster to better determine the impact of constituent psychosomatic syndromes on affected individuals.

### 4.3. Psychosomatic Syndromes and Health Behaviors

The second aim of this study was to identify potential associations between psychosomatic syndromes or profiles and health behaviors.

Contrary to our hypotheses, minimal non-significant associations between psychosomatic syndromes or profiles and health behaviors were observed, as evidenced by very low explained variance values. The exploratory analyses examining associations between syndrome count and health behaviors demonstrated similarly limited explanatory power. These findings suggest that other variables not included in these models may be more relevant determinants of health behaviors in IBD patients.

Few studies have evaluated potential associations between DCPR syndromes and health behaviors, and to our knowledge, this is the first to assess these relationships in IBD patients. A previous study by Gostoli et al. [58], observed associations between dietary habits and persistent somatization, and between smoking and allostatic overload, in patients undergoing colorectal cancer screening. However, their analytical approach differed substantially from the present study, examining only individual associations between specific health behaviors and single psychosomatic syndromes.

Despite the overall null findings, specific psychosomatic profiles and syndromes demonstrated potential associations with health behaviors, which could be explored in future analyses with larger sample sizes. For example, patients in the “Pure Alexithymia” cluster showed reduced odds of being non-smokers (*p* = 0.033), corresponding to approximately 3.85 times higher smoking odds compared to the reference group (patients with no DCPR syndromes or rare syndromes). Notably, no significant association was observed between alexithymia and smoking in the individual syndrome model, indicating that this association is primarily evident when alexithymia occurs without additional psychosomatic syndromes. However, subsequent FDR correction rendered this result non-significant (*p* = 0.400); therefore, these findings should be interpreted cautiously. The relationship between alexithymia and smoking has been documented in literature [88,89,90,91,92], although specific studies remain limited and the underlying mechanisms appear inconclusive. Nevertheless, smoking cessation is strongly recommended for IBD patients according to British Society of Gastroenterology consensus guidelines [93]. Beyond general health risks, smoking is associated with increased disease flare risk, surgical intervention, and post-surgical recurrence in Crohn’s disease [93].

Exploratory sensitivity analyses including psychopharmacological treatment as covariates revealed that the association between Pure Alexithymia cluster membership and smoking became slightly stronger when adjusting for anxiolytic use (*OR* = 0.22 vs. 0.26). Notably, anxiolytic use itself showed a significant association with smoking (*OR* = 0.12, *p* = 0.036), though the direction of causality remains unclear as anxiety disorders themselves—for which anxiolytics are prescribed—may be independently associated with higher smoking rates [94]. However, the primary finding regarding alexithymia-smoking association lost statistical significance after FDR correction; therefore, these results should be interpreted cautiously and primarily serve to inform future research.

Future studies should further characterize the alexithymia-smoking relationship in adequately powered samples, accounting for potential confounders including psychiatric comorbidities and their treatment, and evaluate whether alexithymia-targeted interventions might indirectly impact smoking behaviors. Should these preliminary observations be confirmed, targeted smoking cessation interventions for patients with this cluster profile could be considered when clinically indicated.

On the same vein, patients in the “Overwhelmed Type A Behavior” cluster demonstrated approximately one-third the odds of engaging more frequently in physical activity compared to the reference group (*p* = 0.018). However, subsequent FDR correction rendered this result non-significant (*p* = 0.430), requiring cautious interpretation. The literature provides consistent evidence that physical activity reduces burnout experiences, particularly the exhaustion component [95]. Therefore, the reduced physical activity frequency observed in this cluster may contribute to maintaining the combination of psychosomatic syndromes that characterize it. Notably, regular physical activity can improve well-being and quality of life in IBD patients [96] and is specifically recommended for IBD patients receiving corticosteroids to enhance bone density [93]. Consequently, future studies should focus on evaluating the potential existence of this association and, if confirmed, the effectiveness of targeted interventions to promote physical activity for patients in this cluster, to improve quality of life and physical status, potentially providing indirect benefits for the constituent psychosomatic syndromes.

Finally, associations were identified between irritable mood, type A behavior, and alcohol consumption frequency. Specifically, IBD patients with irritable mood demonstrated approximately one-third the odds of consuming alcohol more frequently (*p* = 0.035), while patients with type A behavior showed nearly double the odds of frequent alcohol consumption (*p* = 0.030). However, subsequent FDR correction rendered these results non-significant (*p* = 0.210), requiring cautious interpretation. The negative association between irritable mood and alcohol consumption frequency was unexpected, as this relationship has not been extensively explored in the literature. While this association likely represents a statistical artifact given the loss of significance after FDR corrections, some studies have highlighted the role of irritability in predisposing individuals to interpersonal alcohol-related aggression [97,98]. If this association were to be confirmed in future studies, one potential explanation could be that, given that irritable mood, according to DCPR-R criteria, involves unpleasant behavioral outbursts not followed by catharsis [6], patients with this syndrome may recognize that alcohol consumption potentially increases their hostility toward others and consequently tend to avoid it.

Regarding Type A behavior, positive associations with alcohol consumption have been reported [99,100], although this evidence is outdated and should be interpreted carefully. Nevertheless, alcohol consumption in IBD patients has been associated with increased inflammation, gastrointestinal symptoms, and pharmacotherapy interference [101]. Given that patients with Type A behavior constituted nearly one-third of the present sample, if a significant association is found in future research, additional studies should further investigate the relationship between this psychosomatic condition and alcohol consumption, and evaluate whether Type A behavior-targeted interventions could reduce alcohol consumption.

### 4.4. Strenghts and Limitations

The conclusions of this study should be interpreted in light of several important limitations. The modest sample size may have limited detection of significant associations with small effect sizes. The absence of a control group and the cross-sectional design restrict the strength and generalizability of these findings. Given that this secondary analysis was conducted on an already existing dataset, some of the included measures may not represent the gold standard or the best methods for evaluating some constructs. For example, the IBD severity covariate used in this study, while based on clinically relevant variables documented in the literature, does not constitute a validated severity index and may be less comprehensive than specific severity indices commonly used in IBD research. Similarly, the lifestyle assessment questionnaire did not permit comprehensive evaluation of dietary habits or physical activity, potentially overlooking specific relevant aspects. Furthermore, the composite dietary score demonstrated very low internal consistency reliability (ω = 0.347), suggesting this measure may inadequately represent dietary patterns in IBD patients. Although consecutive recruitment was employed to minimize bias, no additional procedures were implemented to ensure sample representativeness. Additionally, the clustering structure should be considered exploratory, requiring replication and validation studies to establish generalizability. Finally, following false discovery rate correction, all initially significant results lost statistical significance, indicating potential type I errors. Therefore, these findings should be interpreted with considerable caution and viewed as hypothesis-generating for future research.

This study also presents several important strengths. To our knowledge, this is the first attempt to comprehensively characterize psychosomatic profiles in IBD patients, establishing a foundation for future research and highlighting the importance of considering these aspects in clinical management. Additionally, this represents the first study to systematically evaluate relationships between DCPR syndromes and lifestyle behaviors as a primary objective. Finally, the cluster-based approach to categorizing IBD patients according to psychosomatic syndromes patterns offers potential for early recognition of common profiles that may require targeted interventions or enhanced monitoring for associated risk factors in clinical practice.

## 5. Conclusions

The findings of this study provide an initial characterization of psychosomatic syndromes in IBD patients during remission and their clustering into complex psychosomatic profiles. Approximately 70% of the sample presented at least one DCPR syndrome, indicating widespread prevalence of these conditions in IBD patients. Furthermore, six distinct profiles were identified with specific psychosomatic characteristics that suggest differential risk factors and treatment requirements. Finally, although requiring cautious interpretation due to multiple testing corrections, preliminary associations between syndromes, profiles, and specific health behaviors were observed. Collectively, these findings underscore the importance of comprehensive psychosomatic assessment in IBD patients, even during phases of low disease activity. Moreover, the identification of specific psychosomatic phenotypes and their potential behavioral associations emphasizes the need for personalized treatment approaches that integrate these factors and their clinical implications.

## Figures and Tables

**Table 1:** Sociodemographic characteristics.

Sex	*n*	%
Female	67	60.4
Male	44	39.6
**Education Level**	***n***	**%**
Post-graduate diploma	2	1.8
Master’s degree	19	17.1
Bachelor’s degree	7	6.3
High school	40	36.0
Middle school	38	34.2
Primary school	5	4.5
**Marital Status**	***n***	**%**
Divorced	13	11.7
Married	67	60.3
Single	29	26.1
Widowed	2	1.8
**Age Group**	***n***	**%**
20–29	12	10.8
30–39	25	22.5
40–49	20	18.0
50–59	28	25.2
60–69	19	17.1
70–79	7	6.3

**Table 2:** Clinical characteristics.

IBD Severity	*n*	%
Not severe	96	86.5
Severe	15	13.5
**Years of Disease**	***n***	**%**
0–4	33	29.7
5–9	23	20.7
10–14	24	21.6
15–19	9	8.1
20+	22	19.8
**IBD-Related medications**	***n***	**%**
Mesalazine	93	83.8
Corticosteroids	7	6.3
Azathioprine	5	4.5
Biologicals	6	5.4
**Other medications**	***n***	**%**
Antidepressants	11	9.9
Anxiolytics	7	6.3
Antiplatelets/Anticoagulants	4	3.6
Antidyslipidemics	5	4.5
Antidiabetics	2	1.8
Antihypertensives	15	13.5
**IBD-Related Surgery**	***n***	**%**
No	108	97.3
Yes	3	2.7

IBD: Inflammatory Bowel Disease.

**Table 3:** Lifestyle behaviors.

Smoking Status	*n*	%
Non-smoker	81	73.0
Smoker	30	27.0
**Alcohol Consumption**	***n***	**%**
Never/Occasionally	79	71.2
1–2 times per week	15	13.5
Everyday	15	13.5
More than once a day	2	1.8
**Frequency of Physical Activity**	***n***	**%**
Never/Occasionally	54	48.6
1–2 times per week	32	28.8
Everyday	15	13.5
More than once a day	10	9.0
**Intensity of Physical Activity**	***n***	**%**
Low	44	39.6
Moderate	43	38.7
High	19	17.1
Very High	5	4.5
**Vegetables**	***n***	**%**
Never/Occasionally	21	18.9
2–3 times per week	28	25.2
Once a day	34	30.6
More than once a day	28	25.2
**Fish**	***n***	**%**
Never/Occasionally	57	51.4
2–3 times per week	43	38.7
Once a day	10	9.0
More than once a day	1	0.9
**White Meat**	***n***	**%**
Never/Occasionally	19	17.1
2–3 times per week	64	57.7
Once a day	22	19.8
More than once a day	6	5.4
**Red Meat**	***n***	**%**
Never/Occasionally	59	53.2
2–3 times per week	43	38.7
Once a day	7	6.3
More than once a day	2	1.8
**Fruit**	***n***	**%**
Never/Occasionally	23	20.7
2–3 times per week	22	19.8
Once a day	31	27.9
More than once a day	35	31.5
**Dairies**	***n***	**%**
Never/Occasionally	43	38.7
2–3 times per week	32	28.8
Once a day	27	24.3
More than once a day	9	8.1
**Processed meat**	***n***	**%**
Never/Occasionally	66	59.5
2–3 times per week	30	27.0
Once a day	14	12.6
More than once a day	1	0.9

**Table 4:** DCPR syndromes.

DCPR Diagnosis	Total (*n* = 111)		UC (*n* = 71)		CD (*n* = 40)	
	*n*	%	*n*	%	*n*	%
Absent	34	30.6	17	23.9	17	42.5
Present	77	69.4	54	76.1	23	57.5
**DCPR Syndrome**	***n***	**%**	***n***	**%**	***n***	**%**
Persistent Somatization	7	6.3	4	5.6	3	7.5
Irritable Mood	12	10.8	8	11.3	4	10.0
Type A Behavior	29	26.1	22	31.0	7	17.5
Demoralization	15	13.5	10	14.1	5	12.5
Alexithymia	29	26.1	20	28.2	9	22.5
Allostatic Overload	29	26.1	17	23.9	12	30.0
Thanatophobia	1	0.9	0	0.0	1	2.5
Conversion Symptoms	0	0.0	0	0.0	0	0.0
Health Anxiety	4	3.6	3	4.2	1	2.5
Illness Denial	3	2.7	3	4.2	0	0.0
Anniversary Reaction	1	0.9	1	1.4	0	0.0
Secondary Somatic Symptoms	1	0.9	1	1.4	0	0.0
Disease Phobia	3	2.7	1	1.4	2	5.0
Hypochondriasis	1	1.4	1	1.4	0	0.0

DCPR: Diagnostic Criteria for Psychosomatic Research; UC: ulcerative colitis; CD: Crohn’s disease.

**Table 5:** Validation indices for *k* = 2:6 clustering solutions over six psychosomatic syndromes after removal of patient ID 15 (*n* = 75).

*k*	*ASW*	*CH*	Gap (*SE*)
2	0.230	7.54	0.169 (0.024)
3	0.265	12.61	0.273 (0.023)
4	0.420	19.68	0.387 (0.024)
5	0.512	26.26	0.441 (0.025)
6	0.547	27.99	0.527 (0.026)

*ASW*: Average Silhouette Width; *CH*: Calinski–Harabasz index.

**Table 6:** Final patient distribution (*n* = 75) and cluster characteristics for *k* = 6 solution.

	Cluster 1 (*n* = 7, 9.3%)	Cluster 2 (*n* = 14, 18.7%)	Cluster 3 (*n* = 20, 26.7%)	Cluster 4 (*n* = 9, 12%)	Cluster 5 (*n* = 10, 13.3%)	Cluster 6 (*n* = 15, 20%)
Alexithymia	1 (14.3%)	0 (0%)	20 (100%)	2 (22.2%)	0 (0%)	6 (40%)
Allostatic Overload	0 (0%)	14 (100%)	3 (15%)	9 (100%)	3 (30%)	0 (0%)
Demoralization	0 (0%)	0 (0%)	0 (0%)	3 (33.3%)	10 (100%)	2 (13.3%)
Irritable mood	7 (100%)	0 (0%)	1 (5%)	1 (11.1%)	3 (30%)	0 (0%)
Type A Behavior	5 (71.4%)	0 (0%)	0 (0%)	9 (100%)	0 (0%)	15 (100%)
Persistent Somatization	0 (0%)	1 (7.1%)	3 (15%)	1 (11.1%)	1 (10%)	0 (0%)
*ASW*	0.522	0.890	0.666	0.397	0.398	0.437

**Table 7:** Validation indices for *k* = 2:5 clustering solutions over five psychosomatic syndromes.

*k*	*ASW*	*CH*	Gap (*SE*)
2	0.207	11.70	0.158 (0.026)
3	0.233	10.88	0.282 (0.027)
4	0.401	18.71	0.434 (0.027)
5	0.548	31.01	0.532 (0.028)

**Table 8:** Final patients distribution (*n* = 75) and cluster characteristics for *k* = 5 solution.

	Cluster 1 (*n* = 8, 10.7%)	Cluster 2 (*n* = 20, 26.7%)	Cluster 3 (*n* = 20, 26.7%)	Cluster 4 (*n* = 10, 13.3%)	Cluster 5 (*n* = 17, 22.7%)
Alexithymia	1 (12.5%)	20 (100%)	0 (0%)	0 (0%)	8 (47.1%)
Allostatic Overload	1 (12.5%)	3 (15%)	20 (100%)	3 (30%)	2 (11.8%)
Demoralization	0 (0%)	0 (0%)	2 (10%)	10 (100%)	3 (17.6%)
Irritable mood	8 (100%)	1 (5%)	0 (0%)	3 (30%)	0 (0%)
Type A Behavior	6 (75%)	0 (0%)	6 (30%)	0 (0%)	17 (100%)
*ASW*	0.466	0.776	0.622	0.441	0.367

**Table 9:** Coefficients of the zero-inflation component of the cluster-based model.

Predictor	Estimate	*SE*	*OR*	95% *CI*	*p*-Value	Adjusted *p*-Value
(Intercept)	1.058	0.413	2.88	[1.28, 6.47]	0.010 *	NA
Irritability	−0.575	0.986	0.56	[0.08, 3.89]	0.560	0.974
Pure Allostatic Overload	0.143	0.777	1.15	[0.25, 5.29]	0.854	0.974
Pure Alexithymia	−1.351	0.635	0.26	[0.07, 0.90]	0.033 *	0.400
Overwhelmed Type A Behavior	0.172	0.905	1.19	[0.20, 7.01]	0.849	0.974
Subclinical Depressive Mood	−0.102	0.930	0.90	[0.15, 5.59]	0.913	0.974
Alexithymic Type A Behavior	0.223	0.773	1.25	[0.27, 5.69]	0.773	0.974
IBD Severity	2.004	1.097	7.42	[0.86, 63.67]	0.068	NA

Note: Odds ratios < 1 indicate increased probability of not being a structural zero (smoker). * Significant values.

**Table 10:** Coefficients of the cluster-based ordered probit model for physical activity frequency.

Predictor	Estimate	*SE*	*OR*	95% *CI*	*p*-Value	Adjusted *p*-Value
Irritability	0.224	0.479	1.25	[0.49, 3.20]	0.640	0.818
Pure Allostatic Overload	−0.272	0.356	0.76	[0.38, 1.53]	0.444	0.791
Pure Alexithymia	−0.002	0.309	1.00	[0.54, 1.83]	0.995	0.996
Overwhelmed Type A Behavior	−1.295	0.548	0.27	[0.09, 0.80]	0.018 *	0.434
Subclinical Depressive Mood	−0.214	0.420	0.81	[0.35, 1.84]	0.610	0.818
Alexithymic Type A Behavior	−0.152	0.343	0.86	[0.44, 1.68]	0.658	0.818
IBD Severity	−0.549	0.354	0.58	[0.29, 1.16]	0.121	NA

Odds ratios < 1 indicate increased probability of engaging less frequently in physical activity. * Significant values.

**Table 11:** Coefficients of the syndrome-based ordered probit model for frequency of alcohol consumption.

Predictor	Estimate	*SE*	*OR*	95% *CI*	*p*-Value	Adjusted *p*-Value
Persistent Somatization	0.746	0.523	2.11	[0.76, 5.89]	0.154	0.370
Irritable mood	−1.223	0.580	0.29	[0.09, 0.92]	0.035 *	0.210
Type A behavior	0.624	0.288	1.87	[1.06, 3.29]	0.030 *	0.210
Demoralization	−0.439	0.405	0.64	[0.29, 1.43]	0.279	0.558
Alexithymia	−0.533	0.302	0.59	[0.32, 1.06]	0.078	0.311
Allostatic Overload	−0.171	0.343	0.84	[0.48, 1.48]	0.550	0.875
IBD Severity	−0.170	0.406	0.84	[0.38, 1.87]	0.676	NA

Odds ratios > 1 indicate increased probability of consuming alcohol more frequently. * Significant values.

**Table 12:** DCPR cluster membership across CD and UC patients.

DCPR Diagnosis	Total (*n* = 75)		UC (*n* = 53)		CD (*n* = 22)	
	*n*	%	*n*	%	*n*	%
Irritability	7	9.3	5	9.4	2	9.1
Pure Allostatic Overload	14	18.7	8	15.1	6	27.3
Pure Alexithymia	20	26.7	13	24.5	7	31.8
Overwhelmed Type A Behavior	9	12.0	6	11.3	3	13.6
Subclinical Depressive Mood	10	13.3	8	15.1	2	9.1
Alexithymic Type A Behavior	15	24.5	13	23.9	2	9.1

## Data Availability

The data presented in this study are available on request from the corresponding author due to privacy and ethical restrictions.

## Supplementary Materials

The following supporting information can be downloaded at: https://www.mdpi.com/article/10.3390/jcm14227944/s1, Figure S1: Colored Dendrogram for Solution *K* = 5; Figure S2: Binary Heatmap for Solution *K* = 5; Figure S3: Comparison of Prevalence of Psychosomatic Syndromes (a) and Cluster Membership (b) between UC and CD Patients.

## Abbreviations

The following abbreviations are used in this manuscript:

IBD	Inflammatory bowel disease
DCPR-R	Diagnostic Criteria for Psychosomatic Research—Revised
CD	Crohn’s disease
UC	Ulcerative colitis
ZINB	Zero inflated negative binomial
ASW	Average silhouette width
CH	Calinski–Harabasz
PPO	Partial proportional odds
HAC	Heteroskedasticity and autocorrelation consistent
DSM	Diagnostic and Statistical Manual of Mental Disorders
ICD	International Classification of Diseases
FGID	Functional gastrointestinal disorders
FDR	False discovery rate
IBS	Irritable bowel syndrome
DBT	Dialectical behavior therapy
