# Supplementary material for: Psychosomatic Profiles and Their Association with Health Behaviors in Patients with Inflammatory Bowel Disease (IBD) and Low Disease Activity
Source: J Clin Med. 2025 Nov 9;14(22):7944. doi: 10.3390/jcm14227944 (PMC12653361; doi:10.3390/jcm14227944)
Supplement: Supplementary file 1 [file jcm-14-07944-s001.zip › jcm-3886793-supplementary.pdf]

Supplementary Materials

Clustering solution with 5 syndromes – k = 5

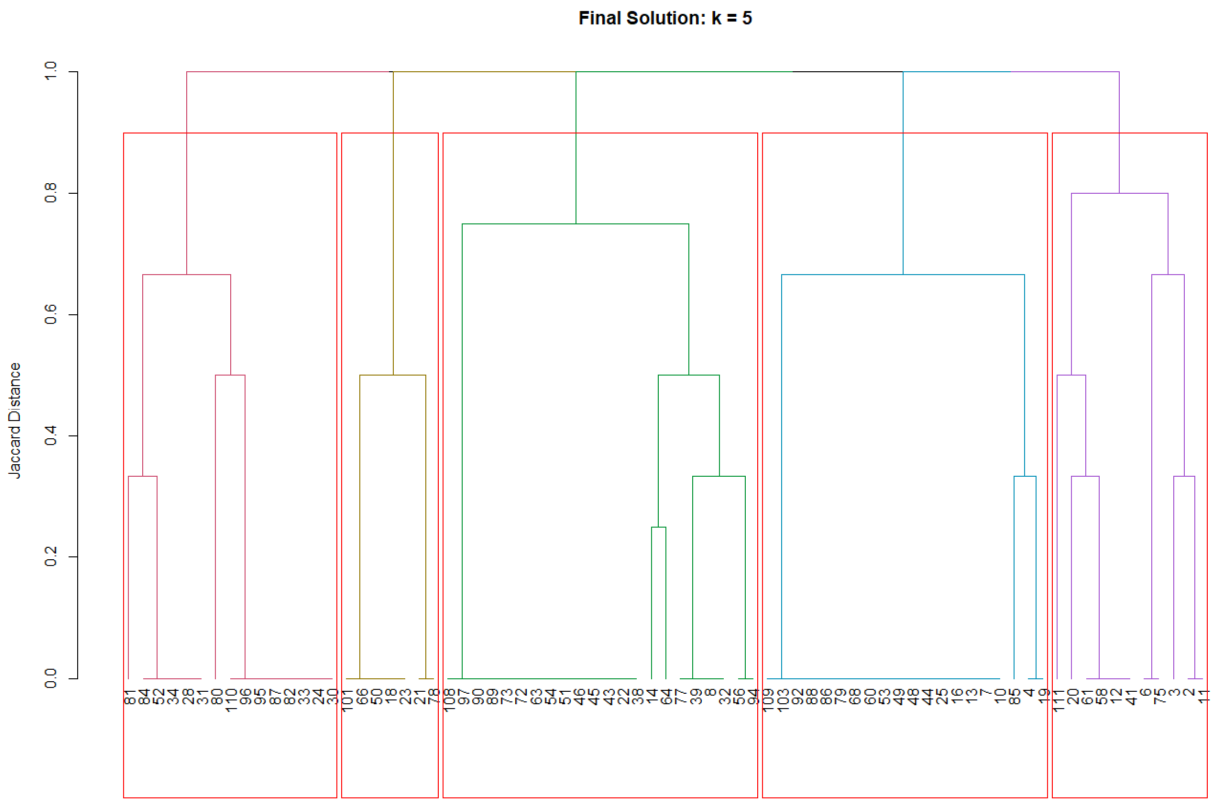

Figure S1. Colored dendrogram for solution k = 5. Colored branches represent the five identified clusters, with each color corresponding to a distinct patient cluster. The horizontal red line indicates the cut height used to obtain the final five-cluster solution.

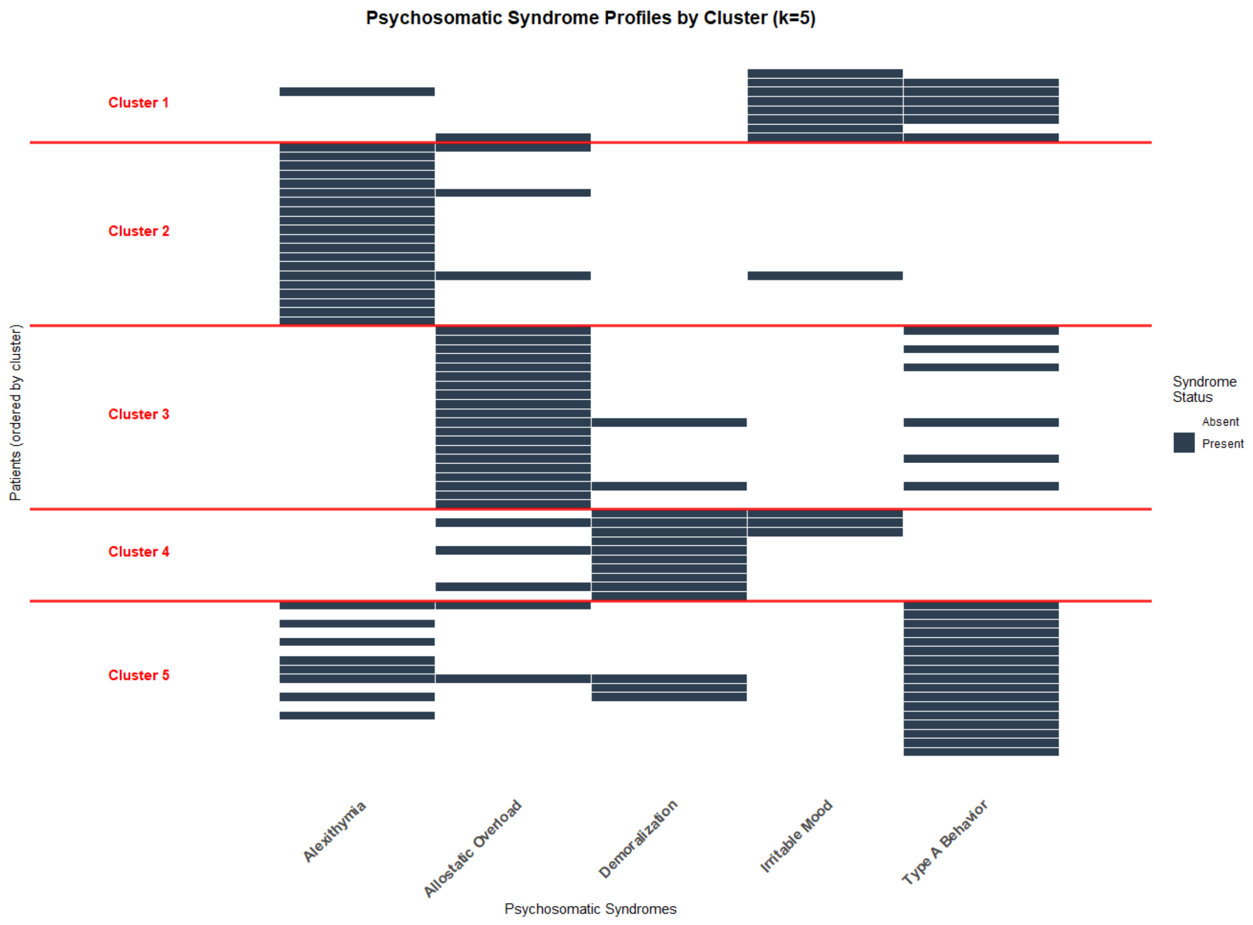

Figure S2. Binary heatmap for solution $k = 5$

Exploratory Analyses

(a)

(b)

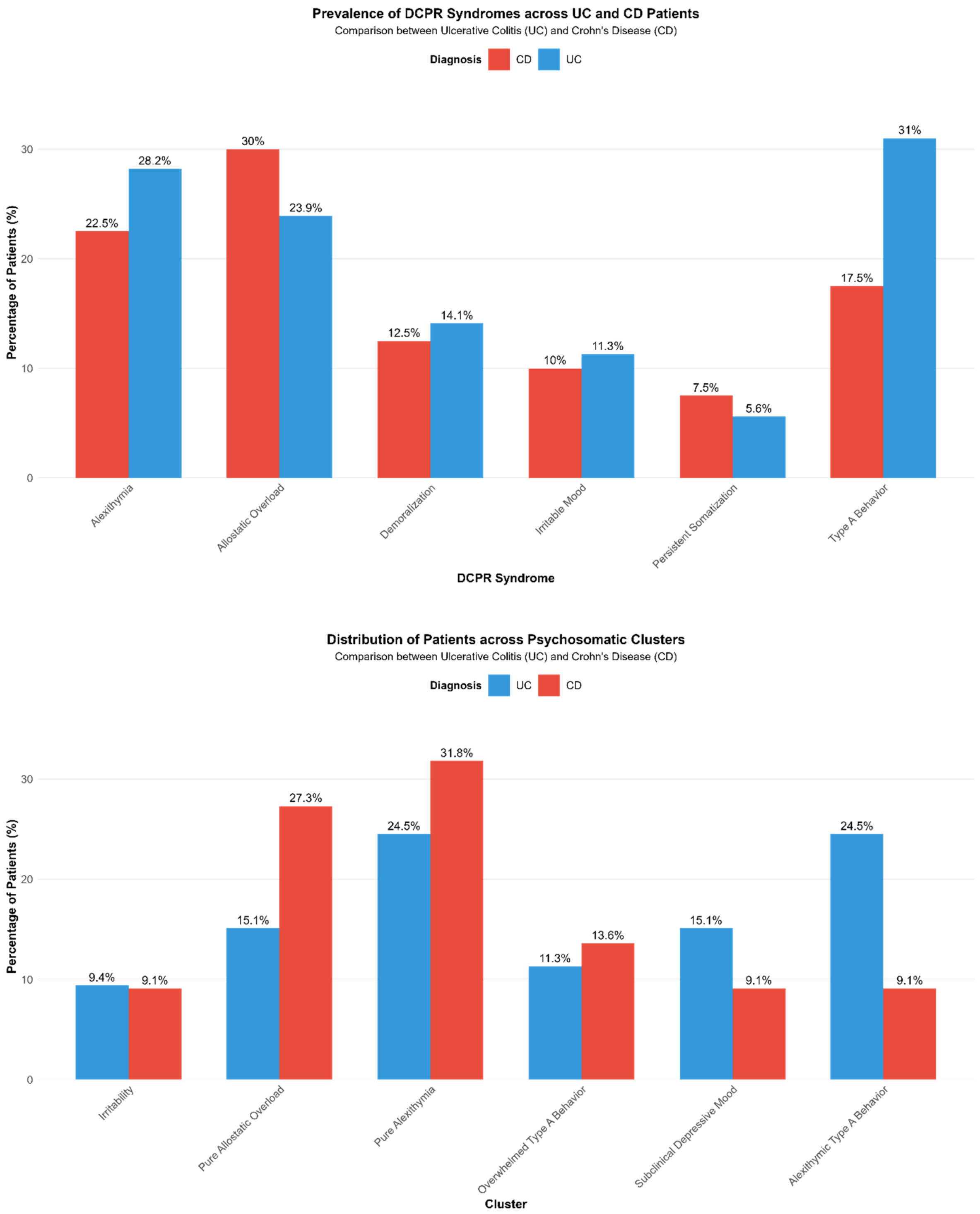

Figure S3. Comparison of prevalence of psychosomatic syndromes (a) and cluster membership (b) between UC and CD patients.
